# Case Report: *DICER1* mutation-associated anaplastic sarcoma of the kidney in a child

**DOI:** 10.3389/fonc.2026.1863816

**Published:** 2026-09-25

**Authors:** Meng Li, Guoqian He, Xia Guo, Ju Gao, Cheng-qi Shen

**Affiliations:** 1 Department of Pediatrics, West China Second University Hospital (WCSUH)-Tianfu, Sichuan Provincial Children's Hospital, Meishan, Sichuan, China; 2 Department of Pediatrics, West China Second University Hospital, Sichuan University, Chengdu, Sichuan, China

**Keywords:** anaplastic sarcoma of the kidney, case report, childhood cancer, DICER1 mutation, renal sarcoma

## Abstract

*DICER1* syndrome is a hereditary tumor predisposition syndrome caused by pathogenic germline variants in *DICER1* and is characterized by multi-organ susceptibility; sarcomas remain uncommon within this spectrum. We report a 2-year-old girl with anaplastic sarcoma of the kidney (ASK). Molecular testing identified a germline *DICER1* c.3300dupA frameshift variant (p.S1101Ifs*3), inherited from her father, and a tumor-associated c.5125G>A missense variant (p.D1709N). The child had neonatal hypothyroidism and language developmental delay. She underwent complete tumor resection followed by 27 weeks of chemotherapy using a Stage I clear cell sarcoma of the kidney (CCSK)-based regimen. At the most recent follow-up, she was 3 years off therapy with no evidence of tumor recurrence or distant metastasis. This case adds to the limited clinicopathologic and molecular descriptions of *DICER1*-associated ASK and supports integrated histopathologic and molecular evaluation, familial *DICER1* assessment, genetic counseling, and long-term syndrome-directed surveillance.

## Introduction

*DICER1* is an evolutionarily conserved cytoplasmic endoribonuclease belonging to the RNase III family. It catalyzes the cleavage of double-stranded RNA to generate mature microRNAs (miRNAs) and small interfering RNAs (siRNAs), and participates in RNA interference and gene-expression regulation (1). Germline or somatic variants in *DICER1* can disrupt miRNA biosynthesis and downstream signaling pathways, thereby contributing to tumorigenesis and selected developmental abnormalities (2). *DICER1* syndrome is a tumor predisposition disorder, most often involving children and young adults, in which germline loss-of-function variants may be accompanied by tumor-specific second hits, particularly hotspot missense variants in the RNaseIIIb domain (3, 4). Core and well-recognized manifestations include pleuropulmonary blastoma, cystic nephroma, ovarian Sertoli-Leydig cell tumor, thyroid nodular disease/cancer, pituitary blastoma, and pineoblastoma; renal tumors, including rare anaplastic sarcoma of the kidney (ASK), have also been reported (5–8). Reported non-neoplastic or developmental features include macrocephaly, ocular abnormalities, and neurodevelopmental findings in selected patients, although speech delay and hypothyroidism are not defining features of classic DICER1 syndrome (4, 9). ASK is rare and is increasingly recognized within the DICER1-related renal tumor spectrum. This article reports a 2-year-old girl with ASK harboring a familial germline *DICER1* c.3300dupA (p.S1101Ifs*3) frameshift variant and a tumor-associated RNase IIIb c.5125G>A (p.D1709N) variant.

## Case report

A 2-year-old girl was admitted to our hospital due to a painless mass in the right abdomen. She had neonatal hypothyroidism treated with long-term levothyroxine replacement therapy and language developmental delay. Abdominal contrast-enhanced computed tomography (CT) revealed a large soft tissue mass measuring 9.7 cm × 10.7 cm in the right renal region, with clear boundaries, heterogeneous density, and septal enhancement after contrast administration, compressing the surrounding organs. Wilms tumor was considered preoperatively; initial staging showed disease confined to the right kidney, with no abdominal lymph node involvement or lung/distant metastasis identified (**Figure 1**). Subsequently, the child underwent right nephrectomy combined with partial resection of the right adrenal gland. Pathological examination showed cystic lesions accompanied by extensive subepithelial spindle cell proliferation. Immunohistochemical staining indicated that epithelial components were positive for PCK, EMA, and PAX-8, while negative for WT-1. Spindle cells were positive for desmin, partially positive for WT-1 and TLE-1, and partially positive for P53, with a Ki-67 proliferation index of 20%–30%. With parental consent, molecular testing was performed as part of the diagnostic evaluation. Testing identified a germline *DICER1* c.3300dupA (p.S1101Ifs*3) frameshift variant and a tumor-associated c.5125G>A (p.D1709N) missense variant. Genetic testing of her father confirmed the same *DICER1* c.3300dupA frameshift variant, while no clinical manifestations of *DICER1* syndrome or relevant tumors have been identified in him to date. Based on the above findings, the child was diagnosed with anaplastic spindle cell sarcoma of the kidney (*DICER1*-associated ASK, Stage I). After surgery, she received chemotherapy according to a Stage I CCSK-based protocol selected by multidisciplinary discussion because no standardized ASK-specific protocol exists. She completed a full 27-week course without severe adverse reactions. At the most recent follow-up, she was 3 years off therapy with no evidence of tumor recurrence or distant metastasis. Chest CT and head CT revealed no evidence of other *DICER1*-related lesions. Thyroid function has been monitored regularly during follow-up. The family also received genetic counseling. No pathogenic *DICER1* variants were detected in the patient’s mother or elder sister. The family pedigree and Sanger sequencing validation are shown in **Figure 2**. Her thyroid function remained stable with replacement therapy, and language development continued to be monitored clinically. A clinical timeline summarizing the episode of care is presented in **Figure 3**.

**Figure 1:**
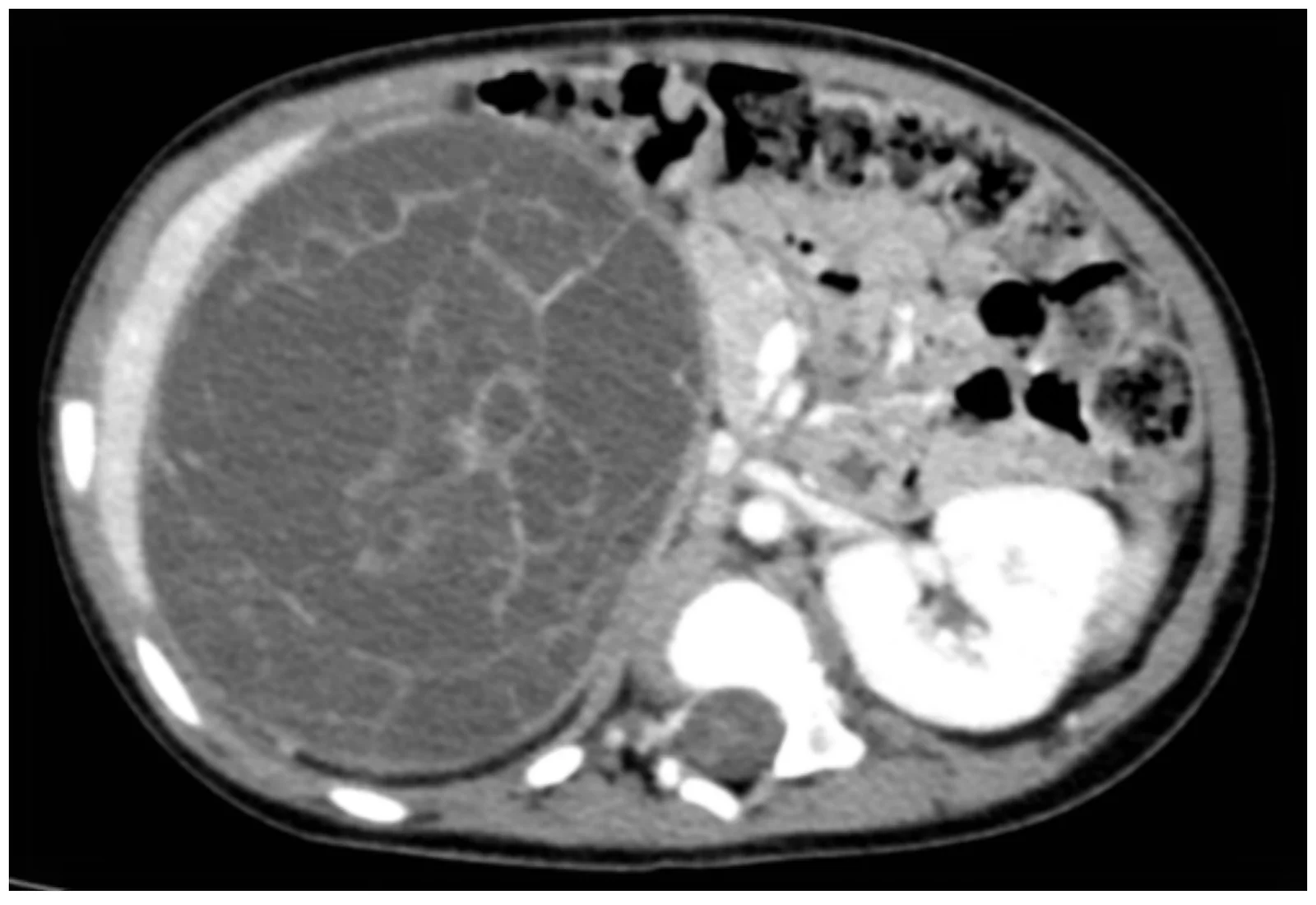
Abdominal contrast-enhanced CT image of the child. A large soft tissue mass (9.7 cm × 10.7 cm) is visible in the right renal region.

**Figure 2:**
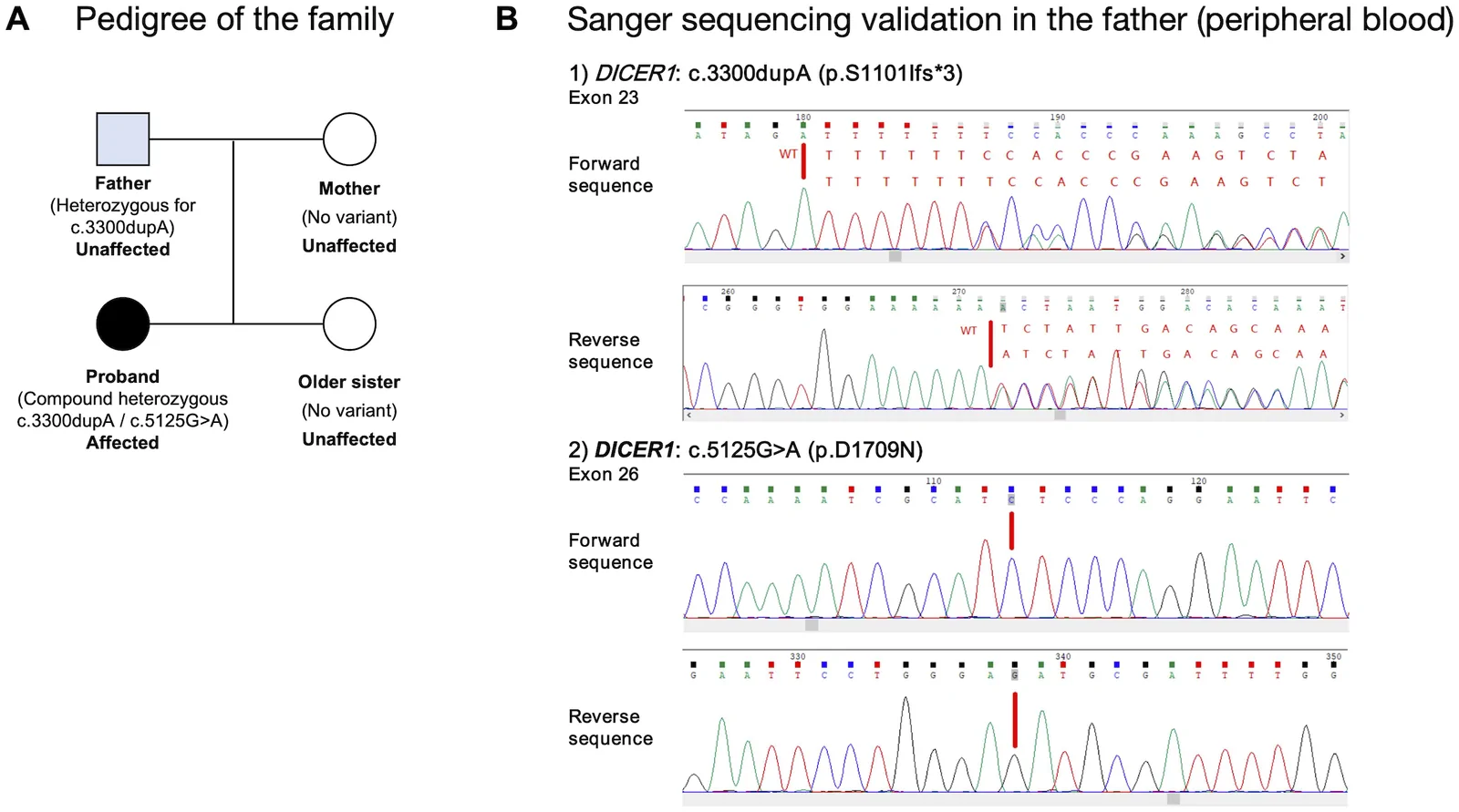
Pedigree and Sanger sequencing validation. **(A)** Pedigree of the family. **(B)** Sanger sequencing validation in the father’s peripheral blood.

**Figure 3:**
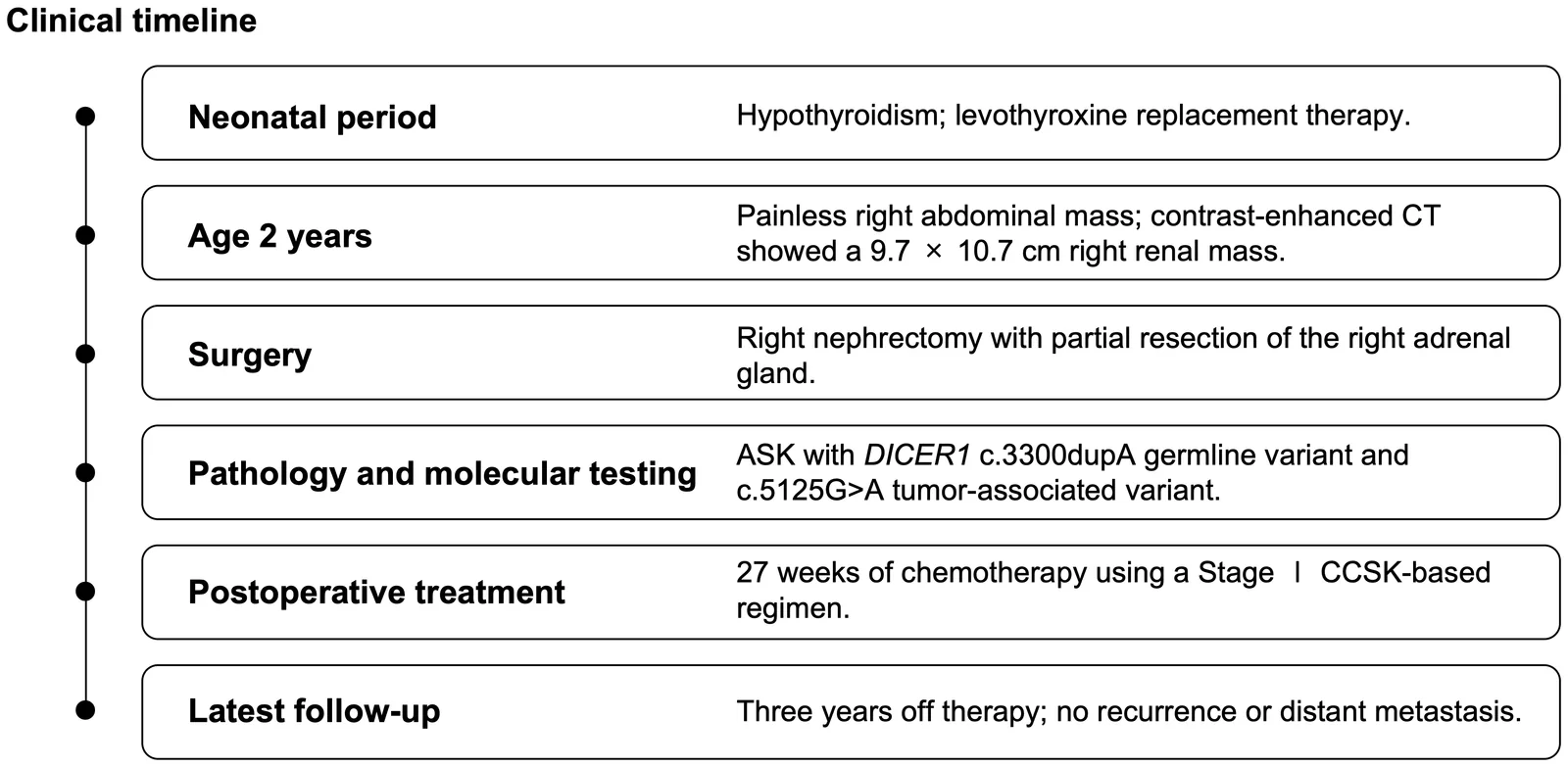
Clinical timeline summarizing presentation, diagnostic work-up, surgery, molecular evaluation, adjuvant chemotherapy, and follow-up.

### Patient perspective

The patient perspective was represented by information from her guardians during follow-up. According to follow-up documentation, the child was clinically well after completion of therapy, with no evidence of tumor recurrence or distant metastasis 3 years off therapy and no severe treatment-related adverse events. Continued oncologic surveillance, thyroid-function monitoring, language-development follow-up, and DICER1 syndrome-directed genetic counseling were discussed with the family.

## Discussion

*DICER1* syndrome is an autosomal dominant hereditary tumor predisposition syndrome caused by germline *DICER1* variants with variable penetrance. The carcinogenic mechanism is commonly explained by a modified two-hit tumor-suppressor model: an inherited or somatic loss-of-function variant affects one allele, and a second somatic event, often a hotspot missense variant in the RNase IIIb domain, alters the remaining allele and contributes to tumor formation (4, 9, 10). In the present case, the paternal c.3300dupA (p.S1101Ifs*3) frameshift variant supports a familial germline *DICER1* loss-of-function variant, whereas the c.5125G>A (p.D1709N) variant lies within the RNase IIIb domain and is compatible with a tumor-associated second hit. Therefore, this case is best described as familial germline *DICER1* predisposition with an additional tumor-associated RNase IIIb variant. This distinction is clinically relevant because it supports genetic counseling and targeted testing of at-risk relatives.

Patients with *DICER1* syndrome require syndrome-directed surveillance because multiple organs may be affected over time. Surveillance recommendations emphasize age-adapted chest imaging for pleuropulmonary blastoma risk, renal/abdominal imaging, thyroid examination or ultrasound, and clinical evaluation guided by symptoms, together with genetic counseling for relatives (4, 9). In the present case, follow-up included imaging surveillance for recurrence/metastasis and thyroid monitoring; genetic counseling and testing of at-risk relatives were recommended. The child’s hypothyroidism and language delay were recorded as coexisting clinical features. Because speech delay is not a classic defining manifestation of *DICER1* syndrome and improvement during follow-up may reflect age-related development or supportive care, these findings should be interpreted cautiously and not as evidence of a treatment-related effect.

The pathological differential diagnosis of pediatric renal tumors requires careful integration of morphology, immunophenotype, clinical information, and molecular findings. ASK is diagnosed primarily on histopathological and immunohistochemical features, while *DICER1* testing supports molecular classification and syndrome recognition but should not be regarded as the sole diagnostic criterion. In this case, cystic lesions with subepithelial spindle cell proliferation and the absence of the classic Wilms tumor triphasic pattern supported ASK. Wilms tumor is usually recognized by blastemal, epithelial, and stromal components and typically shows WT1 positivity, whereas ASK is characterized by spindled/undifferentiated sarcomatous proliferation and does not show the classic Wilms tumor pattern (6, 11). Identification of *DICER1* variants helped integrate the pathological diagnosis with a tumor predisposition syndrome and prompted familial risk assessment.

There is no standardized treatment protocol for *DICER1*-associated ASK, and no targeted therapy for *DICER1* itself has been established. Complete surgical resection combined with adjuvant chemotherapy has been used in reported cases (12). Because ASK is rare and overlaps clinically with other pediatric renal sarcomas, treatment is generally individualized by multidisciplinary pediatric oncology teams; in our case, a Stage I CCSK-based regimen was selected after complete resection because no ASK-specific protocol was available. Recent registry data on anaplastic sarcoma of the kidney/*DICER1*-sarcoma of the kidney suggest that prognosis is influenced by tumor stage, completeness of resection, metastatic disease, and relapse pattern, with more favorable outcomes in localized tumors managed with complete resection and systemic chemotherapy (7). Review articles and recent case reports similarly emphasize integrated pathology-molecular diagnosis, family counseling, and prolonged surveillance rather than *DICER1*-targeted therapy (8, 13). Spinelli et al. reported a multicenter surgical cohort of seven patients with *DICER1* syndrome and compared these cases with 132 published cases from a systematic literature review (14). In that study, ASK was observed in one of seven institutional cases and in only three cases in the reviewed literature, supporting the rarity of ASK within the *DICER1*-associated tumor spectrum (14). Importantly, renal involvement was present in four of the seven institutional patients, and none had a diagnosis of *DICER1* syndrome when renal disease was first detected; the authors also noted that *DICER1* diagnosis often followed surgical treatment (14). These observations are directly relevant to the present case, in which the renal mass was initially suspected to be Wilms tumor and molecular testing after nephrectomy was essential not only for tumor classification but also for recognition of a hereditary tumor-predisposition syndrome. The surveillance approach described by Spinelli et al., including chest and renal imaging, thyroid assessment, and age-adapted follow-up for other syndrome-associated lesions, further supports the need for long-term multisystem surveillance and genetic counseling in the present case (14). The present patient had localized Stage I disease, complete resection, no distant metastasis, and no recurrence 3 years after completion of therapy; nevertheless, the follow-up duration remains limited and ongoing surveillance is required.

## Conclusion

*DICER1*-associated ASK is a rare pediatric renal sarcoma that may clinically mimic Wilms tumor, but diagnosis should rest on integrated morphologic, immunohistochemical, clinical, and molecular features rather than *DICER1* testing alone. The present case illustrates the pattern of an inherited *DICER1* loss-of-function variant with an additional RNase IIIb missense variant, highlights the value of family counseling/testing and syndrome-directed surveillance, and documents no evidence of disease 3 years after completion of therapy following complete resection and adjuvant chemotherapy. Because evidence remains limited, additional well-documented cases with long-term follow-up are needed to refine prognostic factors and management.

## Data Availability

The original contributions presented in the study are included in the article/**Supplementary Material**. Further inquiries can be directed to the corresponding author/s.
