# Supplementary material for: Case Report: DICER1 mutation-associated anaplastic sarcoma of the kidney in a child
Source: Front Oncol. 2026 Sep 25;16:1863816. doi: 10.3389/fonc.2026.1863816 (PMC13650536; doi:10.3389/fonc.2026.1863816)
Supplement: Supplementary file 1 [file Table1.docx]

***Supplementary Material***

# Specimen and Clinical Context

| **Field** | **De-identified information** |
| --- | --- |
| Patient | Pediatric female patient |
| Specimen type | Surgical resection specimen. |
| Anatomic site | Right kidney, with submitted right ureteral tissue/margin, renal hilar fat, and right adrenal tissue. |

# Gross Description

| **Specimen** | **Gross findings from the de-identified report** |
| --- | --- |
| Right ureteral tissue | Tubular tissue with fibroadipose tissue, approximately 2.2 cm in length and 0.6 cm in diameter. A gray-brown intraluminal nodule measuring approximately 1.3 x 0.7 x 0.5 cm was described; the nodule was readily separated from the wall, and the underlying mucosa appeared smooth. |
| Renal hilar fat | Fibroadipose tissue measuring approximately 5.0 x 4.3 x 0.5 cm. No definite nodules or lymph nodes were grossly identified. |
| Right adrenal tissue | Adrenal tissue measuring approximately 2.1 x 1.0 x 0.3 cm and weighing approximately 0.25 g; the surface was smooth. |
| Right renal tumor / nephrectomy specimen | A right kidney with a small amount of perirenal adipose tissue, measuring approximately 15.0 x 7.5 x 6.0 cm. The ureteral stump measured approximately 3.0 cm in length and 0.5-1.1 cm in diameter. The renal capsule was smooth. On sectioning, most of the renal parenchyma was occupied by a mass with multicystic and partially solid areas. The cystic component was gray-white to red, with cysts approximately 0.5-3.0 cm in diameter and clear fluid contents. The wall thickness was approximately 0.1 cm. The partially solid component measured approximately 8.0 x 6.0 x 5.5 cm, was gray-white to gray-yellow and soft, and had an indistinct border with the multicystic component. The tumor compressed the surrounding renal tissue, the renal pelvis was not clearly identifiable, and gross extension into the ureter was described. |

# Microscopic Diagnosis and Initial Pathology Comment

- Right kidney resection specimen: cystic lesion with subepithelial spindle-cell proliferation; further ancillary workup was recommended.
- Ureteral surgical margin: no tumor involvement identified.
- Right ureteral tissue: chronic mucosal inflammation, stromal edema, and focal inflammatory granulation tissue formation.
- Renal hilar fat: no tumor involvement identified.
- The initial comment recommended further genetic analysis and additional diagnostic workup.

# Immunohistochemical Findings

| **Component / marker** | **Result** |
| --- | --- |
| Epithelial component: PCK | Positive |
| Epithelial component: EMA | Positive |
| Epithelial component: PAX-8 | Positive |
| Epithelial component: WT-1 | Negative |
| Spindle-cell component: desmin | Positive |
| Spindle-cell component: WT-1 | Partially positive |
| Spindle-cell component: TLE-1 | Partially positive |
| Spindle-cell component: p53 | Partially positive |
| CD99 | Negative |
| MyoD1 | Negative |
| Myogenin | Negative |
| CD34 | Negative |
| INI-1 | No loss |
| ALK-1 | Negative |
| PanTRK | Equivocal |
| NKX2.2 | Negative |
| Ki-67 | Approximately 20%-30% |
| SMA | Weakly positive |
| MSA | Negative |
| Caldesmon | Negative |

# Differential Diagnosis Listed in the Report

- Wilms tumor with predominant mesenchymal component
- Congenital mesoblastic nephroma
- Clear cell sarcoma of the kidney
- Synovial sarcoma

# Molecular and Ancillary Testing

| **Test** | **Result** |
| --- | --- |
| BCOR internal tandem duplication (BCOR-ITD) | Not detected |
| ETV6/NTRK3 gene fusion | Not detected |
| SS18 gene rearrangement | Not detected |
| DICER1 | Mutation involving exon 23/codon 1709 detected; reported in the manuscript as DICER1 c.5125G>A (p.D1709N). |

# Final Pathological Interpretation

**Final diagnosis from the external pathology report:** DICER1-mutated undifferentiated sarcoma of the kidney. In the manuscript, this pathological entity is discussed as DICER1-associated anaplastic sarcoma of the kidney.

**Clinical recommendation in the report:** The report advised clinical consideration of a possible DICER1 syndrome, including assessment for associated lesions such as pleuropulmonary blastoma when clinically indicated.
